# Liver Fibrosis, Host Genetic and Hepatitis C Virus Related Parameters as Predictive Factors of Response to Therapy against Hepatitis C Virus in HIV/HCV Coinfected Patients

**DOI:** 10.1371/journal.pone.0101760

**Published:** 2014-07-11

**Authors:** Sara Corchado, Luis F. López-Cortés, Antonio Rivero-Juárez, Almudena Torres-Cornejo, Antonio Rivero, Mercedes Márquez-Coello, José-Antonio Girón-González

**Affiliations:** 1 Unidad de Enfermedades Infecciosas, Hospital Universitario Puerta del Mar, Cádiz, Spain; 2 Instituto de Biomedicina de Sevilla, Hospital Universitario Virgen del Rocío/Centro Superior de Investigaciones Científicas/Universidad de Sevilla, Sevilla, Spain; 3 Maimonides Institute for Research in Biomedicine of Cordoba/Reina Sofia University Hospital, Córdoba, Spain; Harvard Medical School, United States of America

## Abstract

**Objective:**

To establish the role of liver fibrosis as a predictive tool of response to pegylated interferon alpha (Peg-IFN) and ribavirin (RBV) treatment in human immunodeficiency (HIV)/hepatitis C virus (HCV) coinfected patients, in addition to recognized predictive factors (HCV load, HCV genotype, IL-28B polymorphism).

**Patients and Methods:**

A sample of 267 HIV/HCV coinfected patients was treated with Peg-IFN and RBV. Predictive factors of rapid (RVR) and sustained (SVR) virological response were analyzed. Independent variables were age, sex, IL28B, −238 TNF-α and −592 IL-10 polymorphisms, HCV genotype, HCV-RNA levels, significant fibrosis or cirrhosis and CD4+ T cell count.

**Results:**

Patients infected by HCV genotype 1 (n = 187) showed RVR and SVR in 12% and 39% of cases, respectively. The parameters associated with RVR were IL28B genotype CC and plasma HCV-RNA levels <600000 IU/ml. Advanced liver fibrosis was negatively associated with SVR in patients without RVR. A SVR was obtained in 42% of subjects with HCV genotype 4, and the independent factors associated with SVR were IL28B genotype CC and an HCV-RNA <600000 IU/ml. A SVR was obtained in 66% of patients with HCV genotypes 2/3; in this case, the independent parameter associated with SVR was the absence of significant liver fibrosis. TNF-α and IL-10 polymorphisms were not associated with SVR, although a significantly higher percentage of −238 TNF-α genotype GG was detected in patients with significant liver fibrosis.

**Conclusions:**

In HIV/HCV coinfected patients with HCV genotypes 1 or 4, RVR, mainly influenced by genotype IL28B and HCV-RNA levels, reliably predicted SVR after 4 weeks of therapy with Peg-IFN plus RBV. In patients infected by HCV genotype 3, an elevated relapse rate compromised the influence of RVR on SVR. Relapses were related to the presence of advanced liver fibrosis. Liver cirrhosis was associated with a −238 TNF-α polymorphism in these patients.

## Introduction

Until recently, hepatitis C virus (HCV) infection has been treated with a combination of pegylated interferon alpha (Peg-IFN) and ribavirin (RBV). In human immunodeficiency virus (HIV) coinfected patients, this treatment attains a sustained virologic response (SVR) in 38–73% of subjects [1]. Recently, the HCV protease inhibitors telaprevir and boceprevir, in combination with Peg-IFN plus RBV, as well as sofosbuvir and simeprevir, have been introduced as treatment for HCV infections [2], [3]. Although the series of HIV-infected patients coinfected by HCV and treated with these direct-acting antivirals has been limited, studies have demonstrated a higher percentage of responses than those obtained with the combination of Peg-IFN plus RBV alone [4], [5], [6]. However, there are several limitations to their use, mainly related to their secondary effects and their pharmacologic interactions with antiretrovirals, occasionally necessitating a change of antiretroviral treatment, which has various clinical limitations [7], [8]. Recently, sofosbuvir and IFN-free regimens have been proven to be efficacious in HIV/HCV coinfection (PHOTON-1 trial), with minimal side effects and drug interactions [9]. However, IFN-containing regimens will still play a role in treatment of HIV-HCV coinfection, especially in resource-poor settings. Consequently, the study of parameters associated with elevated responses to dual therapy (Peg-IFN and RBV combination), which could render the use of telaprevir, boceprevir, sofosbuvir or simeprevir unnecessary, is a key feature of HIV clinical practice.

Parameters influencing the response to Peg-IFN and RBV include, among others, polymorphisms in chromosome 19, near the interleukin 28B (IL28B) gene, in HIV-coinfected patients with infection by HCV genotype 1 [10] or 4 [11], HCV-related factors (infection by HCV genotypes 1 or 4 or higher HCV-RNA levels are associated with a poor response), HIV-related factors (treatment with zidovudine [12] or didanosine [13] increase the rate of adverse events and compromises the response) and liver histopathology (patients with advanced fibrosis or cirrhosis show a decreased percentage of elimination of HCV) [14], [15].

As mentioned above, liver fibrosis stage influences the response to Peg-IFN and RBV, as well as to the new direct-acting antivirals [16]. However, few studies have been conducted on the mechanisms involved in an unfavorable response. Liver fibrosis is influenced by tumor necrosis factor alpha (TNF-α) and interleukin 10 (IL-10). TNF-α stimulates hepatic stellate cells, accelerating liver fibrogenesis [17]. IL-10 is an anti-inflammatory cytokine that downregulates the synthesis of pro-inflammatory cytokines, including TNF-α, and has a modulatory effect on hepatic fibrogenesis [18]. Recently, we demonstrated that single nucleotide polymorphisms at position −238 of the TNF-α gene promoter influences liver fibrogenesis in HIV/HCV coinfected patients: a GG genotype at this position is associated with an independent and significantly higher risk of cirrhosis than GA or AA genotypes [19]. Polymorphism at position −592 of the IL-10 gene promoter has been associated with more accelerated progression of HIV infection [20] and with persistent HCV infection [21]. In the above mentioned series, the CA genotype at position −592 of the IL-10 gene promoter has been associated with a nearly significant higher frequency of liver cirrhosis in bivariate analyses [19].

The objective of this study was to establish the capacity of liver fibrosis, its associated genetic polymorphisms (polymorphism of TNF-α and IL-10 genes) and other well-known parameters influencing the response to Peg-IFN and RBV, to accurately predict SVR in HIV/HCV coinfected patients, grouped as a function of the HCV genotype.

## Patients and Methods

### Patients

Two hundred and sixty seven HIV/HCV coinfected patients consecutively received Peg-IFN and RBV at three university hospitals in Spain from January 2001 to January 2012, and were prospectively followed. All patients were of European ancestry and Caucasian race. The inclusion criteria in this cohort were: 1) coinfection by HIV and HCV; 2) age >18 years; 3) compensated liver disease; 4) no prior anti-HCV treatment; 5) initiation of dual therapy with Peg-IFN plus RBV; and 6) no use of alcohol or illicit drugs for at least one year. In all patients, a whole blood sample was collected and stored at −80°C for subsequent genetic determinations.

All were aged 18–70 years old. All patients were serum negative for hepatitis B surface antigen and for antinuclear, anti-smooth muscle and anti-mitochondrial antibodies. None had genetic iron overload (hemochromatosis) as assessed by serum iron markers, biopsy, and genotyping where indicated. Serum α1 antitrypsin and ceruloplasmin levels were normal.

### Definitions

HIV-infected patients were classified according to the 1993 Centers for Disease Control and Prevention classification of HIV infection. The Spanish AIDS Study Group guidelines (www.gesida.es) were used to indicate the antiretroviral treatment (ART).

Positive serum antibody to HCV and persistent (more than 6 months) plasma HCV-RNA established a diagnosis of chronic HCV infection. Diagnosis of chronic hepatitis or cirrhosis was established according to histological criteria when liver biopsy was performed (n = 161) or by transient elastography (FibroScan®, Echosens, Paris) (n = 267), performed according to a standardized technique. Significant fibrosis was defined as a METAVIR fibrosis score of F3–F4 in liver biopsy or as a liver stiffness (LS) value of ≥8.9 kPa. Cirrhosis was defined as a METAVIR fibrosis score of F4 in liver biopsy or by a liver stiffness (LS) value of >14.6 kPa. Cutoff points of 8.9 and 14.6 kPa were selected according to data validated in HIV/HCV coinfected patients using liver biopsy as reference [22].

### Anti-HCV therapy

All individuals were treated with either Peg-IFN-α2a, at doses of 180 µg per week, in combination with a body weight-adjusted dose of oral RBV (1000 mg per day for patients weighing less than 75 kg, 1200 mg per day for patients weighing more than 75 kg). Patients received 48 weeks of treatment if HCV-RNA was undetectable at week 12; patients with a decrease in plasma HCV-RNA levels >2 log_10_ at week 12 and undetectable HCV-RNA at week 24 received 72 weeks of treatment. At weeks 12 and 24, Peg-IFN-α and RBV were discontinued in non-responders. The scheduled visits were at baseline, every 4 weeks during the first 6 months of treatment and every 12 weeks thereafter. In order to evaluate SVR, a visit was also conducted 24 weeks after stopping therapy.

Responses were analyzed using an on-treatment approach. Rapid virologic response (RVR) was defined as undetectable plasma HCV-RNA at week 4. A decrease in plasma HCV-RNA >2 log_10_ or below the detection threshold at week 12 was considered to be an early virologic response (EVR). Individuals were considered non-responders if they did not reach at least a 2 log_10_ reduction in HCV-RNA levels at week 12 of treatment or undetectable serum HCV-RNA 24 weeks after beginning therapy. An end-of-treatment response (ETR) was defined as undetectable plasma HCV-RNA at the completion of therapy. Virologic breakthrough was defined as detectable plasma HCV-RNA after week 24 of therapy in patients with previously undetectable HCV-RNA. Sustained virologic response (SVR) was defined as undetectable plasma HCV-RNA 24 weeks after the completion of HCV therapy. Viral relapse (VR) was defined as undetectable plasma HCV-RNA levels at the end of treatment, but detectable at 24 weeks post-treatment.

### Laboratory determinations

HIV-1 infection was diagnosed using an EIA (Abbott Laboratories, North Chicago, IL, USA) and confirmed by New Lav Blot I (Bio-Rad, Marnes La Coquette, France). Plasma HIV-1 viral load was determined by the Cobas Amplicor HIV Monitor (Roche Diagnostics, Basel, Switzerland); the cutoff for undetectable viral load was 50 copies/µl. Blood CD4+ T cell count were determined by flow cytometry (FAC Scan, Becton Dickinson Immunocytometry Systems, San Jose, CA, USA).

Anti-HCV antibodies were detected by 3^rd^ generation ELISA (Abbott Diagnostics, Chicago, USA). Plasma HCV-RNA was detected by quantitative PCR (Amplicor HCV Monitor test; Roche Diagnostics, Basel, Switzerland). HCV genotype was identified by line-probe assay (INNOLiPA HCV; Innogenetics, Ghent, Belgium).

### TNF-α, IL-10 and IL-28B polymorphisms

The −238 TNF-α polymorphism (rs361525) consists of a G to A substitution at position −238 in the proximal promoter of the TNF-α gene. The IL-10 polymorphism (rs1800872) consists of a C to A substitution at position −592 in the proximal promoter of the IL-10 gene. The IL-28B polymorphism (rs129679860) consists of a C to T substitution located 3 kilobases upstream of the IL28B gene. Each polymorphism was genotyped by predesigned Taqman assays (Applied Biosystems, Foster City, CA, EEUU) on DNA isolated from whole blood samples, following the manufacturer's instructions.

### Statistical analysis

Descriptive data were expressed as the median (25–75 interquartile range –IQR-) or as absolute number (percentage). Qualitative variables, including genotype distribution, were compared by the chi-square test or Fisher's exact test when necessary. Quantitative variables were compared using the Mann-Whitney U test or ANOVA when necessary. Pearson's correlation coefficient was used to analyze the association between quantitative variables.

A univariate analysis was performed to determine the association between SVR or RVR and parameters that might have an impact on the response to HCV therapy, and variables presenting a significant association (p<0.05) with SVR or RVR were entered into logistic regression models, where RVR and SVR were the dependent variables. Then, a backward stepwise logistic regression analysis was conducted with *p* values of 0.05 and 0.10 for entry and exit, respectively. The median was used as the cutoff value when continuous variables were categorized, unless otherwise specified. In some analyses and in accordance with the predictive ability demonstrated in previous studies, polymorphisms near the IL28B gene were classified as CC or CT/TT [10], those at position −238 in the TNF gene promoter as GG or GA/AA and those at position −592 in the IL-10 gene promoter as CC or CA/AA [19]. A *p* value of <0.05 was considered significant. Statistical analyses were carried out using the SPSS 15.0 statistical software package (SPSS Inc., Chicago, IL, USA).

It should be noted that the genotypic frequencies observed were close to those expected based on allele frequency calculations, and thus conformed to Hardy-Weinberg equilibrium.

### Ethical aspects

This study was conducted in accordance with the Helsinki Declaration. The project and the model for informed consent were approved by the Hospital Universitario Puerta del Mar (Cádiz, Spain) ethical research committee. Written informed consent was obtained from each participant.

## Results

A total of 267 patients were included in the cohort of patients treated with Peg-IFN and RBV. Premature discontinuation of the therapy because of adverse events occurred in 11 (4%) patients. RVR, ETR and SVR were observed in 55 (21%), 144 (54%) and 113 patients (42%), respectively. Relapses occurred in 31 individuals, representing 21% of the 144 patients with ETR. Distribution of the diverse types of response as a function of HCV genotypes is shown in Table 1. Patient characteristics, distributed as a function of the HCV genotype and of the presence or absence of SVR, are shown in Table 2.

**Table 1:** Response to pegylated interferon-α 2a plus ribavirin of 267 HIV/HCV coinfected patients, grouped as a function of HCV genotype.

	HCV genotype 1 (n = 187)	HCV genotype 4 (n = 53)	HCV genotype 2 and 3 (n = 27)
Undetectable HCV at 4^th^ week (rapid virologic response)	23 (12.2)	12 (22.6)	20 (74.1)
Undetectable HCV at 12th week (early virologic response)	87 (46.5)	26 (49.1)	25 (93.0)
Undetectable HCV at 24th week (late virologic response)	104 (55.6)	24 (45.3)	25 (93.0)
Therapy discontinued due to non-response	83 (44.4)	27 (50.9)	2 (7.0)
Therapy discontinued due to adverse effects	8 (4.3)	3 (5.7)	0 (0)
Patients with 72 weeks of treatment	25 (13.4)	0	0
Undetectable HCV at the end of therapy (end-of-therapy virologic response)	96 (51.3)	23 (43.4)	25 (93.0)
Virologic relapse (refers to patients with end-of-therapy virologic response)	23 (24.0)	1 (4.3)	7 (28.0)
Sustained virologic response	73 (39.0)	22 (41.5)	18 (66.7)

Data are shown as absolute number (percentage).

**Table 2:** Characteristics of HIV-HCV coinfected patients treated with pegylated interferon-α 2a plus ribavirin (n = 267), grouped as a function of HCV genotype and presence or absence of sustained virological response.

Parameter	HCV genotype 1 (n = 187)			HCV genotype 4 (n = 53)			HCV genotype 2 and 3 (n = 27)		
	SVR (n = 73)	No SVR (n = 114)	p	SVR (n = 22)	No SVR (n = 31)	p	SVR (n = 18)	No SVR (n = 9)	p
**Age** (years)	41 (37–47)	41 (36–45)	0,716	40 (37–45)	43 (39–47)	0.075	42 (39–45)	42 (40–45)	0.680
**Sex male** (n, %)	60 (82)	97 (85)	0.684	15 (68)	25 (81)	0.345	16 (89)	9 (100)	0.538
**Plasma HCV-RNA load** (log10 IU/ml)	6.10 (5.31–6.63)	6.31 (5.94–6.74)	0.759	5.33 (4.67–5.91)	5.91 (5.47–6.23)	0.185	5.41 (4.93–5.83)	5.86 (4.88–5.97)	0.516
**Plasma HCV-RNA load >600000 IU/ml** (n, %)	46 (63)	92 (81)	0.010	7 (32)	21 (68)	0.013	4 (22)	5 (56)	0.108
**Liver stiffness** (kPa)	11 (8–21)	11 (9–24)	0.292	8 (7–11)	24 (8–37)	0.047	33 (8–57)	30 (27–32)	0.913
**Significant liver fibrosis** (>8.5 kPa) (n, %)	31 (43)	68 (60)	0.025	5 (23)	16 (52)	0.048	5 (28)	7 (78)	0.037
**Liver cirrhosis** (n, %)	27 (37)	50 (44)	0.365	4 (18)	14 (45)	0.076	5 (28)	7 (78)	0.037
**Nadir CD4+ T cell/mm3**	176 (84–263)	149 (33–255)	0.186	193 (52–344)	94 (57–209)	0.198	257 (136–447)	193 (93–400)	0.922
**CD4+ T cell/mm3 at inclusion**	522 (339–709)	479 (323–672)	0.220	525 (337–742)	454 (303–700)	0.555	398 (250–725)	458 (217–774)	0.558
**Concomitant antiretroviral therapy** (n, %)	70 (96)	109 (96)	1.000	21 (96)	30 (97)	1.000	16 (89)	8 (89)	1.000
**Patients with undetectable HIV load** (n, %)	70 (96)	109 (96)	1.000	21 (96)	30 (97)	1.000	16 (89)	8 (89)	1.000
**SNP IL28B rs12979860** (n, %)			<0.001			<0.001			1.000
CC	43 (59)	36 (32)		11 (50)	1 (3)		14 (78)	7 (78)	
CT/TT	30 (41)	78 (68)		11 (50)	30 (97)		4 (22)	2 (22)	
**SNP TNF-α rs361525** (n, %)			0.661			0.720			0.136
GG	62 (85)	100 (88)		19 (86)	25 (81)		13 (72)	9 (100)	
GA/AA	11 (15)	14 (10)		3 (14)	6 (19)		5 (28)	0 (0)	
**SNP IL-10 rs1800872** (n, %)			0.654			0.773			0.420
CC	40 (55)	58 (51)		7 (32)	12 (39)		8 (44)	6 (67)	
CA/AA	33 (45)	56 (49)		15 (68)	19 (61)		10 (56)	3 (33)	
**RVR** (n, %)	22 (30)	1 (1)	<0.001	12 (55)	0 (0)	<0.001	15 (83)	5 (56)	0.175
**ETR** (n, %)	73 (100)	23 (20)	<0.001	22 (100)	1 (3)	<0.001	18 (100)	7 (78)	0.103
**Virologic relapse** (n, % -refers to patients with ETR-)	0 (0)	23 (100)	<0.001	0 (0)	1 (100)	<0.001	0 (0)	7 (100)	<0.001

Abbreviations: SNP: Single nucleotide polymorphism. RVR: Rapid virologic response. ETR: Virologic response at the end of treatment. SVR: Sustained virologic response.

### Response to anti-HCV therapy in HIV-infected patients coinfected by HCV genotype 1

Patients infected by HCV genotype 1 (n = 187) showed a SVR in 39% (n = 73) of cases. The SVR rate was significantly higher in patients harboring IL28B genotype CC, in those with HCV-RNA levels <600000 IU/ml and in those with non-significant liver fibrosis. The percentage of patients with SVR was also significantly higher among individuals with RVR (Table 2). The positive predictive value (PPV) of SVR after attainment of RVR was 96%.

**Figure 1:**
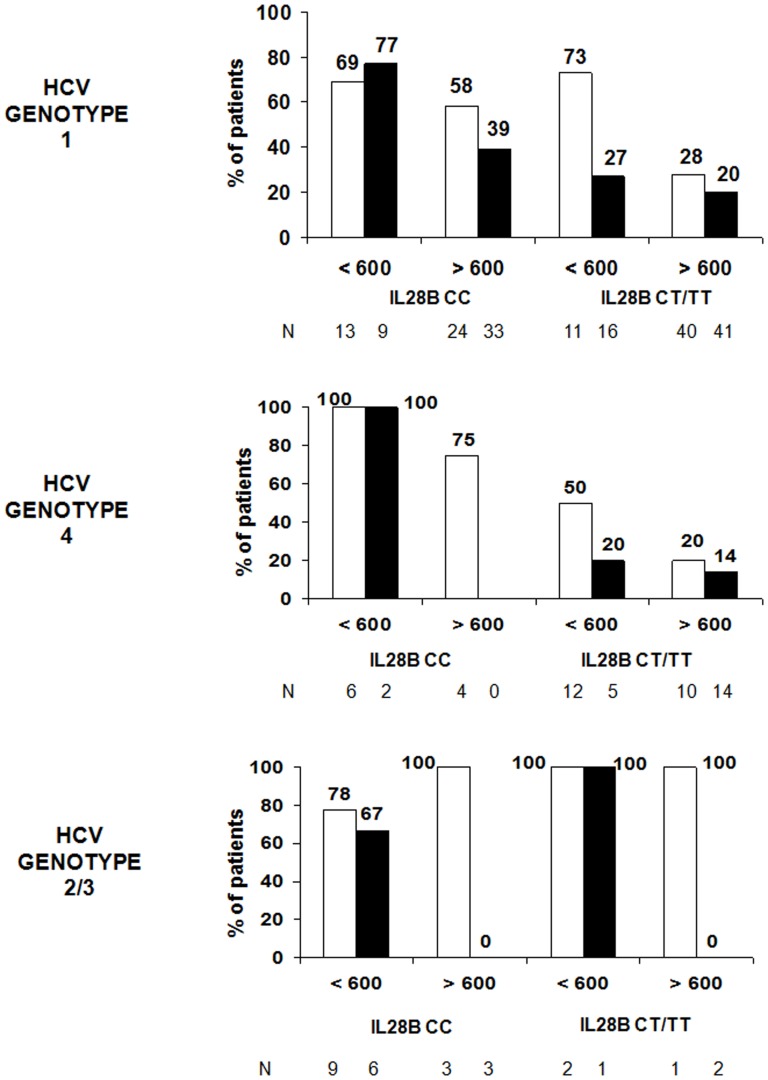
Percentages of HIV/HCV coinfected patients with sustained virological response to pegylated interferon-α 2a plus ribavirin as a function of IL28B polymorphism, HCV-RNA levels and presence (black) or absence (white) of significant liver fibrosis.

When only the baseline parameters were considered (excluding the presence or absence of RVR), the best logistic regression analysis model demonstrated that the baseline characteristics which independently influenced SVR were IL28B genotype CC [Exp(B) 3.30; 95%CI, 1.74–6.25, p<0.001], plasma HCV-RNA levels <600000 IU/ml [Exp(B) 2.56; 95%CI, 1.26–5.20, p = 0.009] and the absence of significant liver fibrosis [Exp(B) 2.15; 95%CI, 1.14–4.05, p = 0.019]. Combining these factors, the probability of a SVR after Peg-IFN and RBV therapy is shown in Figure 1.

**Figure 2:**
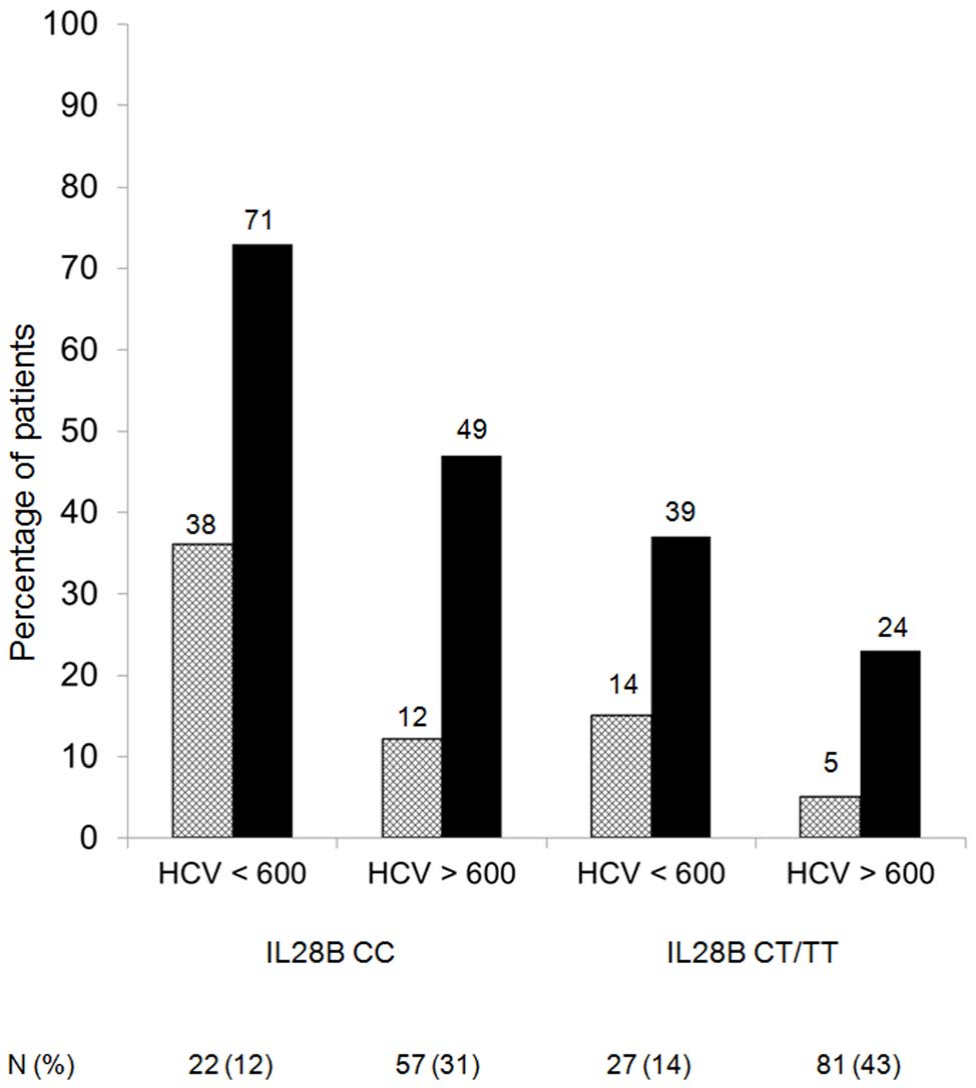
Percentages of HIV/HCV coinfected patients (HCV genotype 1) with rapid (lined) and sustained (black) virological response to pegylated interferon-α 2a plus ribavirin as a function of IL28B polymorphism and HCV-RNA levels.

Because of the importance of RVR as a predictor of SVR, a separate analysis was performed to detect those factors associated with it (Table 2). A RVR was detected in 23 patients (12%). Parameters associated with RVR were IL28B genotype CC and plasma HCV-RNA levels <600000 IU/ml. In the multivariate analysis, both factors were independently associated with RVR: IL28 genotype CC [Exp(B) 2.96; 95%CI, 1.16–7.55, p = 0.023] and HCV-RNA <600000 IU/ml [Exp(B) 3.78; 95%CI, 1.51–9.43, p = 0.004]. Combining these factors, the probability of a RVR is shown in Figure 2.

One hundred and sixty four patients did not show RVR. Of these, 87 had undetectable HCV-RNA at week 12 and were programmed for treatment for 48 weeks, although therapy was discontinued in 8 of them due to adverse effects. Another 25 patients presented with a decrease >2 log10 at week 12 and undetectable HCV-RNA at week 24, and were treated for 72 weeks. Fifty one of 164 patients (31%) without RVR presented SVR (40 of these were treated for 48 weeks and 11 for 72 weeks), and this group included a significantly higher proportion of patients with IL28B genotype CC as well as a higher percentage of patients without significant liver fibrosis (Table 3). In the logistic regression model, only the absence of significant liver fibrosis was independently associated with SVR [Exp(B) 2.67; 95%CI, 1.35–5.30, p = 0.005].

**Table 3:** Characteristics of HIV-infected patients coinfected by HCV genotype 1 treated with pegylated interferon alpha 2a plus ribavirin (n = 187), grouped as a function of the presence or absence of rapid virological response.

Parameter	All patients (n = 187)			Patients without RVR (n = 164)		
	RVR (n = 23)	No RVR (n = 164)	p	SVR (n = 51)	No SVR (n = 113)	p
**Age** (years)	41 (36–45)	41 (37–46)	0.827	41 (38–48)	41 (36–45)	0.527
**Sex male** (n, %)	20 (87)	137 (84)	1.000	41 (80)	96 (85)	0.499
**Plasma HCV-RNA load** (log10 IU/ml)	5.39 (4.90–6.26)	6.31 (5.88–6.74)	0.625	6.25 (5.58–6.78)	6.35 (5.95–6.74)	0.559
**Plasma HCV-RNA load >600000 IU/ml** (n, %)	11 (48)	127 (77)	0.005	35 (69)	92 (81)	0.105
**Liver stiffness** (kPa)	17 (11–21)	10 (8–21)	0.929	9 (8–15)	11 (9–24)	0.221
**Significant liver fibrosis** (>8.5 kPa) (n, %)	14 (61)	85 (52)	0.506	18 (35)	67 (59)	0.007
**Liver cirrhosis** (n, %)	13 (57)	64 (39)	0.119	15 (29)	49 (43)	0.119
**Nadir CD4+ T cell/mm3**	165 (86–208)	151 (51–258)	0.503	189 (82–264)	147 (31–255)	0.236
**CD4+ T cell/mm3 at inclusion**	511 (335–661)	491 (333–688)	0.676	549 (340–757)	477 (320–672)	0.107
**Concomitant antiretroviral therapy** (n, %)	23 (100)	156 (95)	0.599	48 (94)	108 (96)	0.705
**SNP IL28B rs12979860** (n, %)			0.023			0.006
CC	15 (65)	64 (39)		28 (55)	36 (32)	
CT/TT	8 (35)	100 (61)		23 (45)	77 (68)	
**SNP TNF-α rs361525** (n, %)			1.000			0.623
GG	20 (87)	142 (87)		43 (84)	99 (88)	
GA/AA	3 (13)	22 (13)		8 (16)	14 (12)	
**ETR** (n, %)	23 (100)	73 (45)	<0.001	51 (100)	22 (20)	<0.001
**Virologic relapse** (n, % -refers to patients with ETR-)	1 (4)	22 (30)	0.011	0 (0)	22 (100)	<0.001
**SVR** (n, %)	22 (96)	51 (31)	<0.001	51 (100)	0 (0)	

Abbreviations: SNP: Single nucleotide polymorphism. RVR: Rapid virologic response. ETR: Virologic response at the end of treatment. SVR: Sustained virologic response.

### Response to anti-HCV therapy in HIV-infected patients coinfected by HCV genotype 4

Next, we analyzed the probability of SVR in patients coinfected with HCV genotype 4. All of these patients were treated for 48 weeks, and a SVR was obtained in 22 cases (42%). A significantly higher percentage of these patients showed IL28B CC polymorphism and HCV-RNA <600000 IU/ml, and a lower proportion presented significant liver fibrosis (Table 2). When these parameters were associated, the probability of a SVR was that shown in Figure 1. Some 55% of patients with SVR had achieved a RVR. The PPV of a RVR was 100%.

The best logistic regression analysis model demonstrated that the independent factors associated with SVR were IL28B genotype CC [Exp(B) 23.58; 95%CI, 2.54–219.11, p = 0.005] and an HCV-RNA <600000 IU/ml [Exp(B) 4.25; 95%CI, 1.06–17.07, p = 0.042].

### Response to anti-HCV therapy in HIV-infected patients coinfected by HCV genotypes 2 and 3

Lastly, we analyzed the response to anti-HCV treatment of those HIV-infected patients coinfected by HCV genotypes 2 (n = 4) and 3 (n = 23). All of these patients were treated for 48 weeks. Some 74% of patients (20 out of 27) attained a RVR. However, five of them did not show a SVR due to the development of relapses (PPV of RVR: 75%) (Table 2).

Patients with SVR showed a lower percentage of significant liver fibrosis and liver cirrhosis than those without SVR [Exp(B) 9.10, 95%CI 1.39–59.62, p = 0.021]. The remainder of the parameters did not show significant differences.

### Influence of TNF-α and IL-10 polymorphisms on the risk of significant fibrosis or cirrhosis

TNF-α and IL-10 polymorphisms were not associated with SVR. However, the influence of both polymorphisms on an independent prognostic factor of SVR, the presence of significant liver fibrosis or cirrhosis, had been previously demonstrated by our group [19]. Thus, we studied the influence of both polymorphisms on the presence of significant liver fibrosis or cirrhosis in this series (Table 4).

**Table 4:** Characteristics of HIV-HCV coinfected patients treated with pegylated interferon alpha 2a plus ribavirin (n = 267), distributed as a function of the presence or absence of significant liver fibrosis (F3 or F4).

Parameter	Significant liver fibrosis		
	No (n = 135)	Yes (n = 132)	p
**Age** (years)	41 (37–45)	42 (38–46)	0.129
**Sex male** (n, %)	108 (80)	114 (86)	0.192
**HCV genotype**			0.349
1	88 (65)	99 (75)	
2/3	15 (11)	12 (9)	
4	32 (24)	21 (16)	
**Plasma HCV-RNA load** (log10 IU/ml)	6.03 (5.33–6.60)	6.09 (5.62–6.60)	0.193
**Plasma HCV-RNA load >600000 IU/ml** (n, %)	82 (61)	93 (71)	0.122
**Liver stiffness** (kPa)	7 (6–8)	17 (10–28)	<0.001
**Liver cirrhosis** (n, %)	0 (0)	107 (80)	<0.001
**Nadir CD4+ T cell/mm3**	193 (79–281)	146 (55–288)	0.671
**CD4+ T cell/mm3 at inclusion**	511 (370–702)	392 (286–615)	0.092
**Concomitant antiretroviral therapy** (n, %)	127 (94)	127 (96)	0.572
**SNP IL28B rs12979860** (n, %)			0.620
CC	59 (44)	53 (40)	
CT/TT	76 (56)	79 (60)	
**SNP TNF-α rs361525** (n, %)			<0.001
GG	103 (77)	125 (94)	
GA/AA	31 (23)	8 (6)	
**SNP IL-10 rs1800872** (n, %)			1.000
CC	66 (49)	65 (49)	
CA/AA	69 (51)	67 (51)	
**RVR** (n, %)	30 (22)	25 (19)	0.545
**ETR** (n, %)	83 (62)	61 (46)	0.014
**Virologic relapse** (n, % -refers to patients with ETR-)	11 (13)	20 (33)	0.007
**SVR** (n, %)	72 (53)	41 (31)	<0.001

Abbreviations: SNP: Single nucleotide polymorphism. RVR: Rapid virologic response. ETR: Virologic response at the end of treatment. SVR: Sustained virologic response.

IL28B or IL-10 polymorphisms were not associated with significant fibrosis or cirrhosis. Patients with the GG genotype at position −238 in the proximal promoter of the TNF-α gene showed significantly higher values of liver stiffness and a higher proportion of liver cirrhosis than those with genotypes GA/AA. The remaining HCV- or HIV-related characteristics did not show significant differences among patients harboring the TNF-α genotype GG and those with genotypes GA/AA.

## Discussion

Because a successful treatment against chronic HCV infection in HIV-coinfected patients has demonstrated a decrease in liver decompensation events as well as in liver- and non-liver related mortality [23], optimization of this therapeutic strategy is a priority. This study analyzed the efficacy of Peg-IFN and RBV and the factors related to the viral response in a large series of HIV/HCV coinfected patients. A SVR was obtained in 42% of the patients, a percentage similar to that obtained in another series [24]. It should be noted that our study was performed with careful management of side effects in order to attain a low rate of discontinuation due to the toxicity of anti-HCV therapy.

The variables included in our analysis have previously been shown to predict achievement of SVR, although prior attempts to model SVR by combining all these predictors of response in HIV/HCV coinfected patients are scarce [25].

In patients infected by HCV genotype 1, one topic of great interest is the identification of patients with a high probability of response to Peg-IFN and RBV without requiring the inclusion of the direct-acting antivirals telaprevir, boceprevir, sofosbuvir or simeprevir in their treatment. In the particular case of HIV-infected patients, this aspect is important because the interactions with antiretroviral treatment will have to be added to the drawbacks of the new protease inhibitors, along with a higher rate of adverse events and the cost of treating HIV/HCV coinfected patients, which could render universal therapy unaffordable for public health systems [3], [16], [26], [27]. In our study, 39% of HIV-infected patients coinfected by HCV genotype 1 achieved a SVR, similar to other series [10], [15], [24], [28], [29], [30]. One parameter with a high influence on attaining a SVR was the achievement of a RVR: the probability of SVR among patients with RVR was 96%. In HIV/HCV coinfected patients, positive predictive values higher than 75% for HCV genotype 1 have been reported for subjects achieving RVR while receiving therapy [24], [30], [31].

The baseline parameters independently associated with a RVR in HIV-infected patients coinfected by HCV genotype 1 were an IL28B CC genotype and a HCV-RNA <600000 IU/ml. The beneficial impact of the IL28B CC genotype on HCV viral clearance is due to a greater and more rapid HCV viral decline in the first weeks following start of treatment with Peg-IFN plus RBV [31], [32], [33]. In a clinical trial including treatment-naïve, HCV monoinfected patients, subjects carrying the favorable IL28B genotype showed similar rates of SVR, regardless of receiving Peg-IFN and RBV plus boceprevir or placebo (81% vs. 78%, respectively) [33].

It has been proven that the impact of IL28B genotype on the probability of SVR depends on a number of concomitant predictive factors, such as plasma HCV-RNA levels [10], [25], [29], [34]. HCV-RNA levels significantly modify the association between the favorable IL28B genotype and a SVR to Peg-IFN plus RBV in patients infected with HCV genotype 1. Thus, among patients infected by HCV genotype 1 harboring the IL28B genotype CC, 71% of individuals with HCV-RNA <600000 IU/l reached a SVR vs 49% of those with a higher HCV load.

The third independent factor associated with a SVR was the absence of significant liver fibrosis. Explanations about the influence of liver fibrosis are varied: 1) the influence of liver fibrosis could be attributed to the higher number of secondary effects detected in these patients [10], [25]; 2) an alternative explanation is that lower percentages of HCV infected hepatocytes and hepatocyte infection rate are predictors of SVR [35]. Interestingly, the absence of significant liver fibrosis was a predictive parameter of SVR mainly in those patients without RVR. This has a practical consequence: when RVR is used to identify patients to be treated only with Peg-IFN and RBV, the presence or absence of significant liver fibrosis is not a definitive factor in the decision between dual (only Peg-IFN plus RBV) or triple therapy (adding telaprevir or boceprevir).

According to these data, a suitable management strategy for HCV genotype 1 infected patients with IL28B genotype CC and HCV-RNA lower than 600000 IU/ml would be to start a 4-week lead-in phase with Peg-IFN plus RBV followed by the addition or not of boceprevir [36] or telaprevir [37], depending on RVR. This suggestion would need to be studied prospectively.

Another factor to be considered in the parameters associated with a low SVR is the higher rate of relapse after completing a course of therapy. Effectively, a higher relapse rate after completing a course of therapy could contribute to a lower SVR in this population [38]. Relapses were detected in 24 patients (13% of the total population, 21% of those with ETR), within the range seen in other series of coinfected patients treated with Peg-IFN plus RBV (range: 15%–37% of those with ETR) [38], [39]. Relapses were especially present in those individuals without a RVR (only one patient with RVR experienced relapse after suppression of anti-HCV therapy). Among those without RVR, a higher percentage of patients with significant liver fibrosis (higher than 90% of the patients) was demonstrated in patients with relapses, suggesting a role of liver fibrosis in the incidence of relapses and, consequently, in the decreased probability of SVR.

Our study also analyzed the response of HIV-infected patients coinfected by other HCV genotypes. To date, few specific data are available on the treatment of HIV-infected patients coinfected by HCV genotype 4. A previous study by our group showed a SVR of 31% in a series of HIV-infected patients coinfected by HCV genotype 4, with the IL28B genotype being the sole independent prognostic factor of response to Peg-IFN and RBV [11]. In the present study, a SVR was attained in 42% of patients. In our series, patients harboring the IL28B CC genotype and with HCV-RNA levels <600000 IU/l showed a SVR in 100% of cases, although the percentage of patients with both favorable parameters only accounted for 15% of those treated. The probability of response in those with an IL28B CT or TT genotype is very low, especially in those with a higher HCV viral load: those with an IL28B CT/TT genotype and HCV-RNA levels >600000 IU/l showed a SVR in only 17% of cases. Another interesting finding was the importance of achieving a RVR in this group of patients: all patients with a RVR showed a SVR. Liver fibrosis was not an independent parameter with an influence on SVR.

In patients with HCV genotype 2 or 3 coinfection, a SVR was attained in 67% of individuals, a percentage inferior to that observed in other series [24], [39], [40], [41], [42]. The main reason for this result was the percentage of relapses among patients with an ETR. A high proportion of relapses has been observed previously in patients infected by HCV genotype 3 [38], [43], [44]. This has been attributed either to insufficient doses of RBV or to a short duration of treatment (24 weeks) when a RVR had not been achieved [38]. However, in our series, a weight-adjusted dose of RBV was administered and all patients were treated for 48 weeks. Moreover, a RVR had been obtained in 74% of individuals. Infection by HCV genotypes 2 or 3 is a particular case in which the achievement of a RVR is not a good predictor of absence of relapses or of SVR. RVR has obtained a significantly higher PPV of SVR in other series of HIV/HCV patients treated against HCV genotype 3 [24], [40]. The explanation for this discrepancy is probably related to the main predictive factor of relapses and SVR detected in the present study: the existence of significant liver fibrosis. In our series, liver cirrhosis was detected in 44% (12 out of 27 patients) of individuals. Although the importance of this predictive factor was lower in HIV-infected patients coinfected by HCV genotypes 1 or 4, it is evident that liver fibrosis needs to be taken into account in patients coinfected by HCV genotype 3: in these, even in patients with a RVR, a proportion of those with ETR will show higher rates of relapse. Unfortunately, this is not a modifiable factor, except by the possibility of treating patients in an early phase of the liver disease.

The other parameters studied here were polymorphisms of the promoters of TNF-α and IL-10 genes. Our study confirmed that the GG genotype of the TNF-α −238 gene promoter is implicated in the probability of liver cirrhosis, supporting our previous data [19] in a different and larger sample of HIV/HCV coinfected patients. Previous data have demonstrated the impact of TNF-α on liver fibrogenesis: increased concentrations of TNF-α have been detected in the liver of patients with chronic hepatitis C [45] and it has been observed that serum levels of this cytokine are correlated with the histological grading score of hepatitis [46]. Likewise, it has been observed that patients with increased serum levels of TNF-α or its receptors showed a reduced survival rate [47]. In addition, IL-10, an anti-inflammatory cytokine that downregulates the synthesis of pro-inflammatory cytokines, including TNF-α, has a modulatory effect on hepatic fibrogenesis [17], [18]. Moreover, higher serum concentrations of IL-10 have been correlated with SVR after anti-HCV therapy in HCV monoinfected patients [48]. However, the usefulness of TNF-α or IL-10 polymorphisms as predictors of response to Peg-IFN-α plus RBV was not demonstrated in the present series of HIV/HCV coinfected patients.

CD4 T cell counts were over 200/mm3 in the majority of patients, which limits the ability to detect an effect of immune status on the probability of SVR. Previous series [10], [24], [25] did not support an effect of immune status on SVR in HIV/HCV coinfected patients.

In summary, RVR, mainly influenced by IL28B genotype and HCV-RNA levels, can reliably predict a SVR after 4 weeks of therapy with Peg-IFN plus RBV in HIV/HCV coinfected patients with HCV genotypes 1 or 4, which could contribute to reducing unnecessary adverse effects and costs, and to improving the management of these individuals. However, in patients with infection by HCV genotype 3, the elevated relapse rate compromises the influence of RVR on SVR. In patients infected by HCV genotypes 1 and 3, relapses were related to the presence of advanced liver fibrosis.
